# Maladaptive Social Self-Beliefs in Alcohol-Dependence: A Specific Bias towards Excessive High Standards

**DOI:** 10.1371/journal.pone.0058928

**Published:** 2013-03-08

**Authors:** Pierre Maurage, Philippe de Timary, Michelle L. Moulds, Quincy J. J. Wong, Marie Collignon, Pierre Philippot, Alexandre Heeren

**Affiliations:** 1 Laboratory for Experimental Psychopathology, Psychological Science Research Institute, Université catholique de Louvain, Louvain-la-Neuve, Belgium; 2 Department of Psychiatry, Saint-Luc University Hospital, Brussels, Belgium; 3 School of Psychology, University of New South Wales, Sydney, Australia; 4 Department of Psychology, Macquarie University, Sydney, Australia; Radboud University, The Netherlands

## Abstract

**Background:**

Emotional and interpersonal impairments associated with alcohol-dependence have been recently explored, but the distorted cognitive representations underlying these deficits remain poorly understood. The present study aims at exploring the presence of maladaptive social self-beliefs among alcohol-dependent individuals, as these biased self-beliefs have been recently shown to play a crucial role in the development and maintenance of other psychopathological states (social anxiety and depression).

**Methodology/Principal findings:**

Twenty-five recently detoxified alcohol-dependent participants and 25 matched controls filled in self-report questionnaires evaluating maladaptive social self-beliefs, interpersonal problems and several comorbid states (anxiety, social anxiety, depression). As compared to controls, alcohol-dependent individuals showed higher scores than controls for the three subcategories of maladaptive social self-beliefs (high standards, conditional beliefs and unconditional beliefs). Our key finding was that when comorbidities were controlled for, alcohol-dependence was associated with a specific bias towards exaggerated high standards in social contexts. Moreover, these high standards beliefs were strongly correlated with interpersonal problems.

**Conclusions/Significance:**

These results provide the first insights into the influence of cognitive biases on interpersonal problems in addictive states, and suggest that maladaptive self-beliefs could have a central influence on the development and maintenance of alcohol-dependence.

## Introduction

Alcohol-dependence is the most wide spread psychiatric disorder [1] and its deleterious consequences on most body systems, and particularly on the brain, are now largely established [2]. Many studies have explored the behavioral correlates of these cerebral deficits, repeatedly showing impaired performance in a large range of cognitive abilities [3]. In contrast with this extensive exploration of cognition, emotional and interpersonal deficits have long been neglected. While affective and social disturbances constitute central characteristics of alcohol-dependence in clinical settings, they have only been experimentally evaluated in the last decade. Nonetheless, such investigations have already provided clear results. Major emotional alterations have indeed been described among alcohol-dependent subjects (ADS), notably for the decoding of emotional facial expressions [4] and prosody [5], but also for high-level emotional abilities like alexithymia [6], emotional intelligence [7] and empathy [8]. As adapted social interactions are largely reliant on the ability to correctly express and perceive emotional states [9], these emotional deficits increase the social problems frequently observed in alcohol-dependence and favours social isolation [10]. Alcohol consumption is then often augmented to cope with these poor interpersonal relations, initiating a vicious circle [11]. Emotional and interpersonal alterations are thus crucial in the maintenance of alcohol-dependence. Actually, they constitute the main relapse factor after mid-term abstinence [12]. Nevertheless, the core psychological processes underlying these alterations, and particularly the distorted cognitive representations of interpersonal relations, remain poorly understood.

Interestingly, current models of social anxiety and depression [13], [14] postulate that biased cognition plays an important role in the emotional and social difficulties related to these disorders. Anxious or depressed individuals have problematic assumptions about themselves and their social interactions, leading to maladaptive thoughts and behaviors in interpersonal contexts. In the context of social anxiety, these maladaptive self-beliefs have been classified into three categories [13], [15]: (1) Excessively high standards for social performance (e.g., “*I should always have something interesting to say*”), (2) Conditional beliefs concerning the consequences of performing in a certain way (e.g., “*If people get to know me, they won't like me*”), and (3) Unconditional negative beliefs about the self (“*I'm unlikeable*”). Importantly, the intensity of this self-belief bias is directly related to core pathological processes, as it is strongly correlated with the frequency and strength of avoidance behaviors [16], ruminative thoughts [17] and anticipation processes [18] in social anxiety. The extent of conditional and unconditional beliefs biases is also linked with the intensity of depressive symptoms [19]. As a whole, maladaptive self-beliefs significantly contribute to the maintenance of emotional disorders, presumably because of their deleterious influence on affective and interpersonal processes. Extending these observations, we propose that maladaptive self-beliefs may be associated with the affective and social deficits observed in individuals with alcohol-dependence.

Yet, this assumption has not been tested. Several studies have explored self-related cognitions in ADS, and showed that reduced self-esteem [20] and self-efficacy [21], as well as increased self-stigma [22] are characteristics of these individuals. However, biased social cognitions have never been explored in this population: accordingly, this is our goal in the current study. Maladaptive self-beliefs in a social context will be indexed by the Self-Beliefs related to Social Anxiety scale (SBSA) [17], a recently developed tool that evaluates the three categories of self-beliefs postulated by Clark and Wells [13]. Moreover, complementary measures will explore the psychopathological comorbidities (depression, anxiety, social anxiety) and the extent of participants' interpersonal problems.

Our main prediction is that alcohol-dependence is associated with maladaptive self-beliefs, and that their intensity is linked with the extent of interpersonal problems. As comorbid depression or anxiety are frequently observed in alcohol-dependence [23], and as these psychopathological states are associated with maladaptive self-beliefs [13], [16], [18], we also tested their relationship with these biased self-beliefs potentially present among ADS. Finally, we also tested whether alcohol-dependence is associated globally with maladaptive self-beliefs or whether only a specific category of self-beliefs is biased in this disorder.

## Materials and Methods

### Ethics Statement

Participants were provided with full details regarding the aims of the study and the procedure. All participants gave their written informed consent. The study was approved by the Ethical Committee of the Faculty of Medicine (Catholic University of Louvain), and carried out according to the Declaration of Helsinki.

### Participants

Twenty-five inpatients (seven women), diagnosed with alcohol-dependence according to DSM-IV criteria, were recruited during the third week of their treatment in a detoxification center (StLuc Hospital, Brussels, Belgium). Their demographic characteristics appear in Table 1. They had all abstained from alcohol for at least two weeks (mean: 16.07 days; SD 2.46) and were free of any other psychiatric diagnosis as assessed by an exhaustive psychiatric examination. The mean alcohol consumption among patients just before detoxification was 20.58 alcohol units (an alcohol unit corresponding to 10 grams of ethanol) per day (SD 14.69), the mean number of previous detoxification treatments was 0.96 (SD 1.09). Mean duration of alcohol-dependence was 10.84 years (SD 12.32) and mean age at the beginning of alcohol-dependence was 40.16 years (SD 11.19). Patients were matched for age, gender and education with a control group of 25 volunteers (control subjects, CS) who were free of any history of psychiatric disorder or drug/substance abuse, and whose alcohol consumption was lower than 10 alcohol units per week. CS were recruited among the experimenters' acquaintances and the hospital employees, and abstained from any alcohol consumption for at least three days before testing. Exclusion criteria for both groups included major medical problems, neurological disease (including epilepsy), visual impairment, and polysubstance abuse. Each participant had normal-to-corrected vision. Education level was assessed according to the number of years of education completed since starting primary school. CS were free of any medication but 12 ADS still received moderate doses of benzodiazepines (mean in ADS: 28.91 mg/day of Diazepam; SD 35.71). ADS took part in an extensive psychotherapeutic program during their detoxification (individual and group therapy). Participants were not paid for their participation.

**Table 1:** Alcohol-dependent (ADS) and control (CS) subjects' results for demographic and psychological measures: mean (S.D.).

	ADS (N = 25)	CS (N = 25)
**Demographic measures**		
Age^NS^	51(12.19)	50.48(12.17)
Gender ratio (female/male)^NS^	7/18	7/18
Educational level^NS^	15.16(3.8)	15.56(2.58)
**Psychopathological measures**		
BDI***	17.24(11.37)	4.4(4.73)
STAI A**	39.76(11.84)	28.98(8.88)
STAI B***	47.3(10.62)	32.7(10.01)
LSAS*	40.75(25.07)	27.04(15.63)
FNE**	18(9.22)	10.95(7.74)
IIP Total***	1.684(0.57)	0.796(0.52)

NS =  Non-significant;*p<.05;**p<.01;***p<.001.

### Measures

#### The Self-Beliefs related to Social Anxiety scale

(SBSA) [17] is a self-report questionnaire evaluating the strength of beliefs related to the self in social situations. Participants endorsed 15 items in which they rated their level of agreement with self-beliefs on an 11-point Likert scale, from 0 (“Do not agree at all”) to 10 (“Strongly agree”). This questionnaire has strong psychometric properties and includes three categories of maladaptive self-beliefs that can lead to erroneous social behavior: (1) excessively high standards for social performance (HS 4 items, e.g., “I have to appear intelligent and witty”); (2) conditional beliefs concerning social evaluation (CB 7 items, e.g., “If I don't get everything right, I'll be rejected”); (3) unconditional beliefs about the self (UB 4 items, e.g., “People think I'm boring”). The total score is the sum of the all items scores, and each subscale score is the sum of the subscale items scores. The validated French version was used. The SBSA scale has excellent internal consistency in English (Cronbach's α = .94 for global score, .85 for HS, .91 for CB, .82 for UB) and French (Global score:.90, HS:.75, CB:.89, UB:.79).

#### Control measures

Participants also completed the following questionnaires: (1) The State and Trait Anxiety Inventory (STAI A-B) [24], a 40-item questionnaire assessing state (STAI-A) and trait anxiety (STAI-B). The validated French version was used [25]; (2) the Beck Depression Inventory (BDI short-version) [26], a 13-item measure of symptoms of depression. The validated French version was used [27]; (3) the Liebowitz Social Anxiety Scale (LSAS, self-report format) [28], a 24-item scale measuring anxiety and avoidance of social interaction and performance situations. The validated French version was used [29]; (4) the Fear of Negative Evaluation scale (FNE) [30], a 30-item questionnaire evaluating a person's apprehension about negative evaluation. The validated French version was used [31]; (5) the Inventory of Interpersonal Problems (IIP) [32], a 127-item questionnaire evaluating the presence and intensity of interpersonal problems and relational difficulties. The validated French version was used [33].

## Results

### Preliminary analyses: Group equivalence

As shown in Table 1, ADS and CS did not significantly differ for age [*t*(48) = .15, *ns*], gender and education [*t*(48) = .43, *ns*], confirming successful matching of the groups. Nevertheless, alcohol-dependence was associated with significantly higher scores for every psychological measure: depression [*t*(48) = 5.21, *P*<.001], state anxiety [*t*(48) = 3.64, *P*<.01], trait anxiety [*t*(48) = 5.01, *P*<.001], fear of negative evaluation [*t*(48) = 2.93, *P*<.01], social anxiety [*t*(48) = 2.32, *P*<.05], and interpersonal problems [*t*(48) = 5.74, *P*<.001].

### Effect of alcohol-dependence on maladaptive social self-beliefs

**Figure 1:**
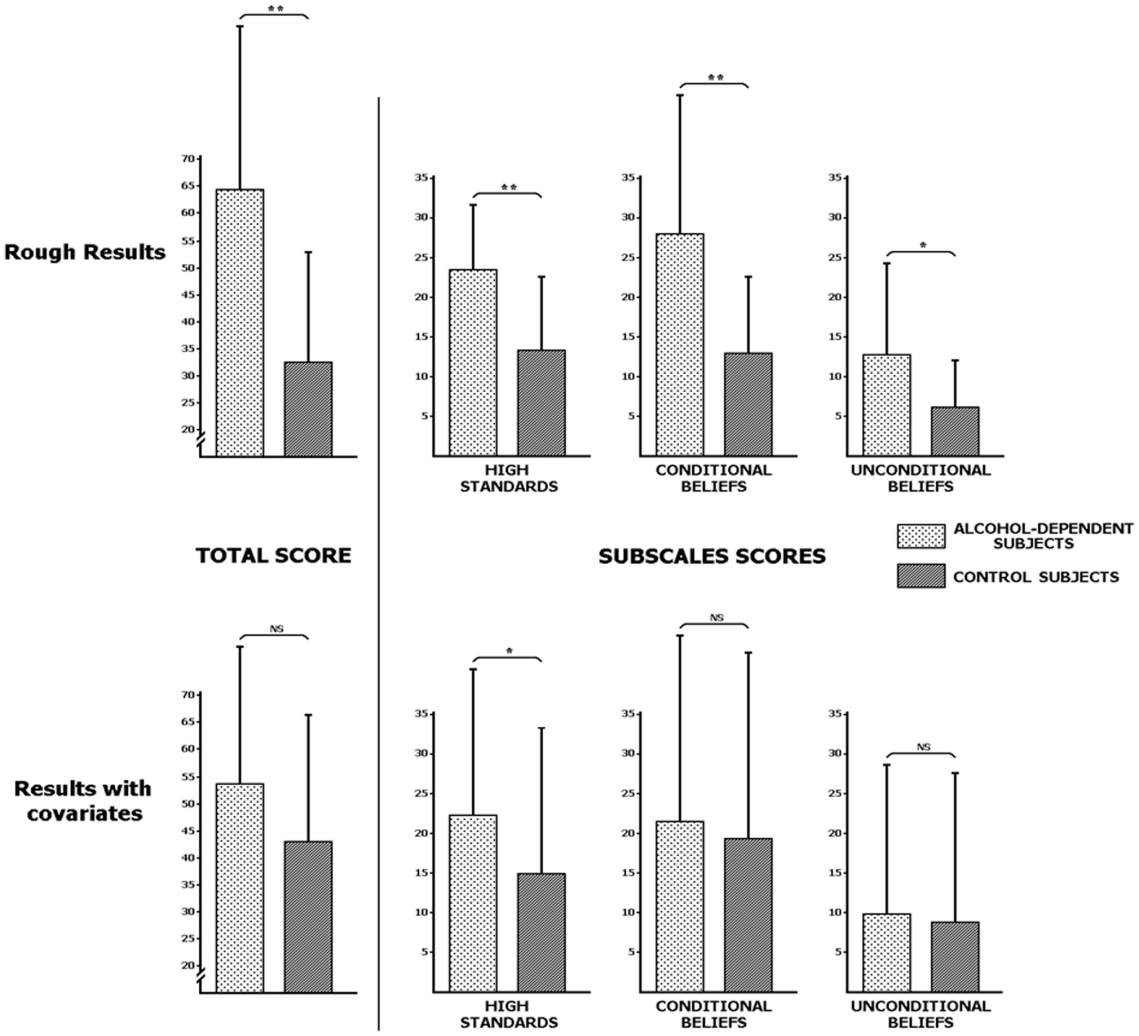
Self-Beliefs Social Anxiety Scale results for each group (alcohol-dependent and control subjects). Global scores are presented on the left and the scores for each subscale (high standards, conditional beliefs and unconditional beliefs) are presented on the right. The upper part depicts rough scores and the lower part depicts the scores corrected for depression (BDI score), anxiety (STAI A and B scores) and social anxiety (FNE and LSAS scores) using covariance analyses. Covariates appearing in the model are evaluated at the following values: 10.82 for BDI, 34.37 for STAI A, 40 for STAI B, 22.89 for LSAS and 14.47 for FNE. NS = Non-significant;*p<.01;**p<.001.

A multivariate analysis of variance (MANOVA) was applied to examine group differences on SBSA subscales, with groups (ADS, CS) as between-subjects factor and subscales (high standards, conditional beliefs, unconditional beliefs) as dependent variables. As illustrated in Figure 1, ADS presented significantly higher scores than CS for SBSA total score [*F*(3,46) = 7.33, *P*<.001, η^2^_p_ = .323] and for each subscale: high standards [*F*(1,48) = 18.54, *P*<.001, η^2^_p_ = .279], conditional beliefs [*F*(1,48) = 14.69, *P*<.001, η^2^_p_ = .234] and unconditional beliefs [*F*(1,48) = 7.71, *P*<.01, η^2^_p_ = .138].

In order to exclude any influence of variance related to comorbid psychopathological states in the self-beliefs results in alcohol-dependence, the psychological measures (i.e., BDI, STAI A-B, FNE and LSAS scores) were introduced as covariates in a second analysis (MANCOVA) with groups (ADS, CS) as between-subjects factor and subscales (high standards, conditional beliefs, unconditional beliefs) as dependent variables. No significant differences were observed for SBSA total score [*F*(3,41) = 2.34, *ns*, η^2^_p_ = .146], conditional beliefs [*F*(1,43) = .45, *ns*, η^2^_p_ = .01] or unconditional beliefs [*F*(1,43) = .12, *ns*, η^2^_p_ = .003], but ADS presented higher scores than CS for the high standards subscale [*F*(1,43) = 7.17, *P*<.01, η^2^_p_ = .143].

### Complementary analyses

#### Links between maladaptive self-beliefs and interpersonal problems

No significant correlation was found between any self-beliefs subscale and interpersonal problems among CS (*r*<.39, *ns*). In alcohol-dependence, no significant correlation was found between interpersonal problems and conditional (*r* = .22, *ns*) or unconditional (*r* = .36, *ns*) beliefs, but high standards were significantly correlated with IIP total score (*r* = .45, *p*<.05).

#### Medication effect

No significant correlation was found between medication level and any questionnaire result in the alcohol-dependent group (all *P*>.05).

## Discussion

While they have been far less explored than cerebral and cognitive alterations in alcohol-dependence, emotional and interpersonal ones are omnipresent in alcohol-dependence and highly involved in relapse [10], [12]. Unfortunately, little is known about the biased representations underlying these affective and social difficulties. As maladaptive beliefs about the self in social contexts influence emotional and social processes in other psychopathological states [16], [34], the present study investigated social self-beliefs in ADS, and their links with interpersonal problems. A main result was that ADS exhibit significantly higher scores than CS for SBSA total and for every subscale, which constitutes the first description of a general bias for maladaptive self-beliefs related to social contexts in addictive states. Nevertheless, similarly biased self-beliefs have been found in general and social anxiety [17], [19], and these psychopathological states are frequently associated with alcohol-dependence, as confirmed by the comorbidities observed in the present sample. These comorbidities were thus taken into account in a second analysis to determine their own impact on maladaptive self-beliefs, aside from the direct effect of alcohol-dependence.

As illustrated in Figure 1, group differences on SBSA global score, conditional beliefs and unconditional beliefs disappeared when these comorbidities were introduced as covariates, but the ADS still presented significantly higher scores for the high standards subscale. Hence, a central observation of the present study is that alcohol-dependence, beyond anxious and depressive comorbidities, is associated with excessively high standards in social contexts. ADS thus seem to particularly overvalue requested behavior in interpersonal context (e.g., “I have to appear intelligent and witty”) and their obligation to constantly have a perfect social behavior (e.g., “I must get everyone's approval”). In other words, they overestimate the level of interpersonal performance needed to obtain the desired positive social outcome. The potential reactions of others if this social performance is not optimal (conditional beliefs) and the general negative opinions of others concerning subject's social behaviors (unconditional beliefs) are also overrated, but these biased cognitions seem to be mostly a result of the comorbid conditions that these patients frequently present with. Nevertheless, we acknowledge that covariate analyses do not allow us to clearly determine what part of variance related to comorbidities is removed [35], and thus in interpreting our covariates analyses we must keep this in mind.

However, it has been recently shown that social anxiety is associated with excessive high standards, conditional beliefs and unconditional beliefs [19]. The present results together with earlier ones thus suggest that, while maladaptive self-beliefs are present in a wide range of psychopathological states (including bulimia nervosa [36] and psychosis [37]) and are thus transdiagnostic, the three categories of self-beliefs could be distinctly distorted in different psychopathological states. Finally, these exaggerated high standards are strongly associated with the social problems encountered in real-life situations. Indeed, while interpersonal problems were not linked with SBSA total score or conditional/unconditional beliefs subscales, high standards scores were highly correlated in the ADS with IIP. No causal inference can be drawn from these correlational results, but they nevertheless confirm that biased cognition in social context is intimately connected with the actual behaviors and performance in these contexts.

These results have important implications. At fundamental level, they offer the first insights into the biased psychological representations involved in the affective and social difficulties described in alcohol-dependence. ADS present massive impairments in emotional and interpersonal situations [10], and this pathology is marked by strong social stigma [38] as well as higher sensitivity to social rejection [39]. The present results showing the links between maladaptive self-beliefs and social deficits could thus constitute a first step towards a thorough exploration of the possible pathways linking biased psychological representations, social alterations and alcohol-dependence. First, an individual who holds excessively high social standards may frequently have difficulties reaching those unrealistic standards in social contexts, and the resulting distress could be temporally reduced by increasing the use of alcohol as a self-medication. Second, individuals with excessively high social standards may believe that alcohol enhances their social abilities, and may thus increase their alcohol consumption to meet their high social standards. Third, problematic alcohol use and related interpersonal difficulties may already be present for an individual. Excessively high social standards may then develop to guide behaviour so as to attempt to avoid interpersonal conflict, and allow alcohol use to continue. The present results are consistent with these pathways but the specific temporal ordering of the variables have to be examined further in future studies. More globally for psychopathology, our results show a significant correlation between maladaptive self-beliefs and the intensity of interpersonal problems in everyday life. This observation should be confirmed and extended among different psychopathological populations, but it offers the first confirmation of the association between maladaptive self-beliefs and self-reported difficulties with social behaviors.

At a clinical level, these results suggest that maladaptive self-beliefs negatively influence social integration in alcohol-dependence and could favour the maintenance of alcohol consumption by reinforcing the vicious circle between interpersonal problems, social isolation and alcohol consumption. Therapeutic interventions reducing this self-beliefs bias might thus interfere with the vicious circle and reduce alcohol consumption or relapse risk. Interventions focusing on maladaptive self-beliefs have been tested in social anxiety [40]. Specifically, “widening the bandwidth” exercises [15] aim at questioning the high standards during social performance and showing that the range of adapted social behaviors is larger than those allowed by these high standards. Proposing these brief exercises during the rehabilitation process might lower the exaggerated high standards present in ADS, and improve social functioning after detoxification.

Several limitations have to be underlined. First, while the results observed are highly significant and based on strong statistical power, the sample size was quite limited. Larger studies should be conducted, particularly to explore the heterogeneity of ADS. Second, inappropriate social behaviors were assessed using self-report. Future studies could usefully examine the associations between self-beliefs and behavioral indices of social behaviors, measured by an experimental approach complementing our questionnaire-based approach (e.g., multimodal assessment of social behaviors [18], [41]). Third, this first exploration in alcohol-dependence should be followed up by explorations of maladaptive self-beliefs in other psychopathological states in which emotional and interpersonal alterations are highly present (e.g., schizophrenia and autism), in order to confirm whether distorted self-beliefs in a social context constitute a transdiagnostic process. More specifically, exploring these self-beliefs in other addictive states would give important insights on the common nature of different addictions. Moreover, the links between these maladaptive self-beliefs and core psychological processes in alcohol-dependence (e.g., rumination, psychological craving, avoidance of social interactions, lack of self-forgiveness [42]) should be further explored to better understand the mediating variables linking the influence of these biased cognitions on social behaviors. Finally, the proposal that these exaggerated high standards hamper treatment seeking should also be tested, as increasing the proportion of patients included in detoxification process is a crucial public health goal in alcohol-dependence [43].

To conclude, this study sought to explore for the first time whether self-beliefs related to social interactions are characteristics of ADS. Our main prediction that alcohol-dependence would be associated with maladaptive self-beliefs was strongly supported, as shown by significantly higher scores in ADS than CS for SBSA global score and subscales. Critically, when the comorbidities (depression, anxiety and social anxiety) were taken into account using covariate analyses, the only remaining significant bias among ADS was the presence of exaggerated high standards during social interactions. Independently from common comorbidities, alcohol-dependence is thus linked with exaggerated high standards in interpersonal contexts, which could contribute to the vicious circle between alcohol consumption, interpersonal problems and social isolation [11]. Indeed, significant correlations were also found between excessive high standards and interpersonal problems, suggesting a direct link between maladaptive self-beliefs and actual social difficulties. While these results should be confirmed and extended in future studies, they nonetheless provide the first insights into the influence of underlying cognitive biases on interpersonal problems in addictive states, and thus open exciting possibilities for future investigations in this area.

## References

[1] Harper C, Matsumoto I (2005) Ethanol and brain damage. Curr Opin Pharmacol 5: 73–78. 15661629 10.1016/j.coph.2004.06.011

[2] McIntosh C, Chick J (2004) Alcohol and the nervous system. J Neurol Neurosurg Psychiatry 75: 16–21. 10.1136/jnnp.2004.045708 PMC1765664 15316040

[3] Pitel AL, Beaunieux H, Witkowski T, Vabret F, Guillery-Girard B, et al (2007) Genuine episodic memory deficits and executive dysfunctions in alcoholic subjects early in abstinence. Alcohol Clin Exp Res 31: 169–178. 10.1111/j.1530-0277.2007.00418.x PMC2895973 17511749

[4] Philippot P, Kornreich C, Blairy S, Baerts I, Den Dulk A, et al (1999) Alcoholics' deficits in the decoding of emotional facial expression. Alcohol Clin Exp Res 23: 1031–1038. 10397287

[5] Monnot M, Lovallo WR, Nixon SJ, Ross E (2002) Neurological basis of deficits in affective prosody comprehension among alcoholics and fetal alcohol-exposed adults. J Neuropsychiatry Clin Neurosci 14: 321–328. 12154157 10.1176/jnp.14.3.321

[6] Taieb O, Corcos M, Loas G, Speranza M, Guilbaud O, et al (2002) Alexithymia and alcohol dependence. Ann Med Interne (Paris) 153: 51–60. 12218885

[7] Cordovil de Sousa M, de Timary P, Cortesi MA, Mikolajczak M, du Roy de Blicquy P, et al (2010) The emotional components of craving: covariation of craving, affect, alexithymia, emotional intelligence in alcohol-dependent patients. Pers Individ Dif 48: 16–21.

[8] Maurage P, Grynberg D, Noël X, Joassin F, Philippot P, et al (2011) Dissociation between affective and cognitive empathy in alcoholism: A specific deficit for the emotional dimension. Alcohol Clin Exp Res 35: 1662–1668. 21599717 10.1111/j.1530-0277.2011.01512.x

[9] Feldman RS, Philippot P, Custrini RJ (1991) Social competence and nonverbal behaviour. In: Feldman RS, editor. Fundamentals of Non-Verbal Behavior. New York, NY: Cambridge University Press. 329–350.

[10] Uekermann J, Daum I (2008) Social cognition in alcoholism: a link to prefrontal cortex dysfunction? Addiction 103: 726–735. 18412750 10.1111/j.1360-0443.2008.02157.x

[11] Kornreich C, Philippot P, Foisy ML, Blairy S, Raynaud E, et al (2002) Impaired emotional facial expression recognition is associated with interpersonal problems in alcoholism. Alcohol Alcohol 37: 394–400. 12107044 10.1093/alcalc/37.4.394

[12] Zywiak WH, Westerberg VS, Connors GJ, Maisto SA (2003) Exploratory findings from the reasons for drinking questionnaire. J Substance Abuse Treat 25: 287–292. 10.1016/s0740-5472(03)00118-1 14693258

[13] Clark DM, Wells A (2010) A cognitive model of social phobia. In: Heimberg RG, Liebowitz MR, Hope DA, Schneier FR, editors. Social Phobia: Diagnosis, Assessment, and Treatment. New York, NY: Guilford Press. 69–93.

[14] Phillips WJ, Hine DW, Thorsteinsson EB (2010) Implicit cognition and depression: a meta-analysis. Clin Psychol Rev 30: 691–709. 20538393 10.1016/j.cpr.2010.05.002

[15] Clark DM (2001) A cognitive perspective on social phobia. In: Crozier WR, Alden LE, editors. International Handbook of Social Anxiety: Concepts, Research and Interventions Relating to the Self and Shyness. New York, NY: John Wiley and Sons. 406–430.

[16] Wong QJJ, Moulds ML (2011a) The relationship between the maladaptive self-beliefs characteristic of social anxiety and avoidance. J Behav Ther Exp Psychiatry 42: 171–178. 21315878 10.1016/j.jbtep.2010.11.004

[17] Wong QJJ, Moulds ML (2009) Impact of rumination versus distraction on anxiety and maladaptive self-beliefs in socially anxious individuals. Behav Res Ther 47: 861–867. 19608157 10.1016/j.brat.2009.06.014

[18] Wong QJJ, Moulds ML (2011b) Impact of anticipatory processing versus distraction on multiple indices of anxiety in socially anxious individuals. Behav Res Ther 49: 700–706. 21821231 10.1016/j.brat.2011.07.007

[19] Wong QJJ, Moulds ML (2011c) A new measure of the Maladaptive self-beliefs in social anxiety: Psychometric properties in a non-clinical sample. J Psychopathol Behav Assess 33: 285–297.

[20] Iorgulescu G (2010) Low self-esteem in women with eating disorders and alcohol abuse as a psycho-social factor to be included in their psychotherapeutic approach. J Med Life 3: 458–464. 21254749 PMC3019074

[21] Trucco EM, Connery HS, Griffin ML, Greenfield SF (2007) The relationship of self-esteem and self-efficacy to treatment outcomes of alcohol-dependent men and women. Am J Addict 16: 85–92. 17453609 10.1080/10550490601184183

[22] Schomerus G, Corrigan PW, Klauer T, Kuwert P, Freyberger HJ, et al (2011) Self-stigma in alcohol dependence: consequences for drinking-refusal self-efficacy. Drug Alcohol Depend 114: 12–17. 20933344 10.1016/j.drugalcdep.2010.08.013

[23] Fein G, Di Sclafani V, Finn P, Scheiner D (2007) Sub-diagnostic psychiatric comorbidity in alcoholics. Drug Alcohol Depend 87: 139–145. 16965876 10.1016/j.drugalcdep.2006.08.009 PMC1880901

[24] Spielberger DC, Gorsuch RL, Lushene R., Vagg PR, Jacobs GA (1983) Manual for the State-Trait Anxiety Inventory. Palo Alto, CA: Consulting Psychology Press.

[25] Bruchon-Schweitzer M, Paulhan I (1993) Adaptation francophone de l'Inventaire d'Anxiété Trait-Etat (Forme Y) de Spielberger. Paris, France: Editions du Centre de Psychologie Appliquée.

[26] Beck AT, Beck RW (1972) Screening depressed patients in family practice: A rapid technique. Postgrad Med 52: 81–85. 4635613 10.1080/00325481.1972.11713319

[27] Collet LL, Cottraux JJ (1986) Inventaire abrégé de la dépression de Beck (13 items): Étude de la validité concurrente avec les échelles de Hamilton et de ralentissement de Widlöcher. Encephale 12: 77–79. 3743520

[28] Cox BJ, Ross L, Swinson RP, Direnfeld DM (1998) A comparison of social phobia outcome measures in cognitive-behavioral group therapy. Behav Modif 22: 285–297. 9670801 10.1177/01454455980223004

[29] Heeren A, Maurage P, Rossignol M, Vanhaelen M, Peschard V, et al (2012a) Self-report version of the Liebowitz Social Anxiety Scale: Psychometric properties of the French version. Can J Behav Sci 44: 99–107.

[30] Watson D, Friend R (1969) Measurement of social-evaluative anxiety. J Consult Clin Psychol 33: 448–457. 5810590 10.1037/h0027806

[31] Douilliez C, Baeyens C, Philippot P (2008) French validation of the Fear of Negative Evaluation Scale and the Social Avoidance and Distress Scale. Rev Franc Clin Comp Cogn 13: 1–12.

[32] Horowitz LM, Rosenberg SE, Baer BA, Ureño G, Villaseñor VS (1988) Inventory of interpersonal problems: Psychometric properties and clinical applications. J Consult Clin Psychol 56: 885–892. 3204198 10.1037//0022-006x.56.6.885

[33] Hansenne M, Charles G, Pholien P, Panzer M (1993) Mesure subjective de l'impact d'un événement: traduction française et validation de l'échelle d'Horowitz. Psychol Med 25: 86–88.

[34] Wong QJJ, Moulds ML (2012) Processing mode during repetitive thinking in socially anxious individuals: evidence for a maladaptive experiential mode. J Behav Ther Exp Psychiatry 43: 1064–1073. 22695223 10.1016/j.jbtep.2012.05.002

[35] Miller GA, Chapman JP (2001) Misunderstanding analysis of covariance. J Abnorm Psychol 110: 40–48. 11261398 10.1037//0021-843x.110.1.40

[36] Bergin JL, Wade TD (2012) A cross-sectional analysis of the cognitive model of bulimia nervosa. Int J Eat Disord 45: 776–786. 22407509 10.1002/eat.22012

[37] Stowkowy J, Addington J (2012) Maladaptive schemas as a mediator between social defeat and positive symptoms in young people at clinical high risk for psychosis. Early Interv Psychiatry 6: 87–90. 21951894 10.1111/j.1751-7893.2011.00297.x PMC3460802

[38] Pescosolido BA, Martin JK, Long JS, Medina TR, Phelan JC, Link BG (2010) ‘A disease like any other’? A decade of change in public reactions to schizophrenia, depression, and alcohol dependence. Am J Psychiatry 167: 1321–1330. 20843872 10.1176/appi.ajp.2010.09121743 PMC4429867

[39] Maurage P, Joassin F, Philippot P, Heeren A, Vermeulen N, et al (2012) Disrupted regulation of social exclusion in alcohol-dependence: an fMRI study. Neuropsychopharmacology 37: 2067–2075. 22510722 10.1038/npp.2012.54 PMC3398714

[40] Goldin PR, Gross JJ (2010) Effects of mindfulness-based stress reduction (MBSR) on emotion regulation in social anxiety disorder. Emotion 10: 83–91. 20141305 10.1037/a0018441 PMC4203918

[41] Heeren A, Reese HE, McNally RJ, Philippot P (2012b) Attention training toward and away from threat in social phobia: Effects on subjective, behavioral, and physiological measures of anxiety. Behav Res Ther 50: 30–39. 22055280 10.1016/j.brat.2011.10.005

[42] Scherer M, Worthington EL, Hook JN, Campana KL (2011) Forgiveness and the bottle: promoting self-forgiveness in individuals who abuse alcohol. J Addict Disord 30: 382–395. 10.1080/10550887.2011.609804 PMC3292781 22026530

[43] Rhem J, Shield KD, Gmel G, Rehm MX, Frick U (2012) Modelling the impact of alcohol dependence on mortality burden and the effect of available treatment interventions in the European Union. Eur Neuropsychopharmacol: In press. 10.1016/j.euroneuro.2012.08.001 22920734

